# Effect of the TiO2 nanoparticles on the selected physical properties of mineral trioxide aggregate

**DOI:** 10.4317/jced.53166

**Published:** 2017-02-01

**Authors:** Mohammad Samiei, Maryam Janani, Naser Asl-Aminabadi, Negin Ghasemi, Baharak Divband, Sajjad Shirazi, Kayvan Kafili

**Affiliations:** 1 Associate Professor, Department of Endodontics, Dental Faculty, Tabriz University (Medical Sciences), Tabriz, Iran; 2 Assistant Professor, Department of Endodontics, Dental and Periodontal Research Center, Dental Faculty, Tabriz University (Medical Sciences), Tabriz, Iran; 3 Professor, Department of Pediatric Dentistry, Dental Faculty, Tabriz University (Medical Sciences), Tabriz, Iran; 4 Assistant Professor, Department of Endodontics, Dental Faculty, Tabriz University (Medical Sciences), Tabriz, Iran; 5 Assistant Professor, Department of Chemistry, Tabriz University , Tabriz, Iran; 6 Research Assistant, Dental Faculty, Tabriz University (Medical Sciences), Tabriz, Iran; 7 Private Practice, Tabriz, Iran

## Abstract

**Background:**

Some of the efforts to improve the properties of Mineral Trioxide Aggregate (MTA) include incorporation of some nanoparticles such as Titanium dioxide (TiO2). The aim of this study was to evaluate the effect of TiO2 nanoparticles on the setting time, working time, push-out bond strength and compressive strength of MTA.

**Material and Methods:**

The physical properties to be evaluated were determined using the ISO 6786:2001 and 9917 specifications. Fifteen samples of each material (MTA or MTA with 1% weight ratio of TiO2 Nanoparticles) were prepared for any evaluated physical property. Data were analyzed using descriptive statistics and T-test. Statistical significance was set at *P*<0.05.

**Results:**

There was the significant effect of the material type (presence and absence of TiO2 nanoparticles) on the push-out bond strength, compressive strength, working time and setting time, with significantly higher values achieved in the group with TiO2 nanoparticles than the group without these particles (*P*=0.01 for the setting time and compressive strength, *P*=0.03 for the working time and *P*=0.001 for the bond strength).

**Conclusions:**

Based on the findings of this *in vitro* study, incorporation of the TiO2 nanoparticles with weight ratio of 1% increased the setting time, working time, compressive strength and push out bond strength of MTA.

** Key words:**Mineral trioxide aggregate, nanoparticles, physical properties, titanium dioxide.

## Introduction

Mineral trioxide aggregate (MTA) has been suggested for use in many challenging endodontic procedures such as apexogenesis, apexification, perforation repair and apical surgery (1,2). However, as a biomaterial, MTA has good properties, including sealing ability, biocompatibility and the capacity to induce tissue regeneration (3). It is not an easy material to handle. The long setting time of MTA makes it unsuitable material for one-appointment treatment (3,4).

Some of the efforts to improve the properties of MTA include incorporation of some nanoparticles such as silver, zinc and titanium dioxide (TiO2), which have drawn attention in the dental materials field (2,5,6). TiO2 nanoparticles have wide industrial applications, such as in pharmaceuticals, pigments, and cosmetics, particularly with a wide range of uses in the biomedical field as an integrating agent to bone tissue (7-11). In a study, incorporation of TiO2 nanoparticles to white Portland cement resulted in an increase in its flexural and compressive strengths and a decrease in its setting time. Given the similarities between MTA and Portland cement (3), it is expected that incorporation of these nanoparticles to MTA would improve its properties.

However, new compositions of MTA or use of various additives can affect MTA’s ideal characteristics, necessitating comprehensive investigations. In this context, it is important to pay attention to the physical properties of the resultant product. One of the properties that should be taken into account in this situation is the compressive strength, which is low in MTA and is considered one of its disadvantages (12). This parameter is very important in some clinical conditions, such as the use of biomaterials in repairing furcal restorations or apexogenesis of the teeth with immature apices (13). Studies carried out on the compressive strength of MTA have shown that hydration accelerators such as calcium chloride, acid etching, blood contamination, fetal bovine serum , and an increase in liquid-to-powder ratio decrease the compressive strength. Incorporation of silica and bismuth oxide nanoparticles has no effect on the compressive strength; however, mixing with chlorhexidine 0.12% (CHX) and mechanical mixing in an amalgamator and placement in association with ultrasonic agitation increase the compressive strength (12,14-19).

The bond strength of MTA to dentin is another important property because it reflects the resistance of the material to displacement in the face of occlusal forces or forces due to the placement of a restorative material on it and is an indirect indication of the material’s seal (20,21). The bond strength of MTA to dentin has been evaluated in different studies by considering the factors affecting the bond strength, including various mixing techniques, the effect of acidic and alkaline environments, the effect of placing phosphate-buffered saline solution on MTA, the effect of contamination with blood and presence or absence of the smear layer (20,22-28).

Proper setting and working times are the prerequisites of a biomaterial. Since the long setting time of MTA does not allow a one-session treatment modality and it is possible for the material to be washed away from the area, several materials have been incorporated into MTA over time to decrease its setting time; for example, 5% calcium chloride, k-y jelly and NaOCl gel have decreased its setting time from 50 minutes to 20-25 minutes (3,4).

Since no studies are available on the effect of incorporating TiO2 nanoparticles into MTA on its properties, the aim of this study was to evaluate the effect of titanium dioxide nanoparticles on some selective physical properties of MTA, including setting time, working time, push-out bond strength and compressive strength.

## Material and Methods

The design of this study was approved in Tabriz Dental and Periodontal Research Center’s investigation committee (No 56-4384). Two groups (n=15) were designed in order to evaluate the physical properties; group 1 consisted of MTA (Angelus, Londrina, Paraná, Brazil) and group 2 consisted of MTA with 1 wt% of TiO2 nanoparticles. In both groups the powder-to-liquid ratio was 3:1.

-Setting Time

This physical property was evaluated based on ISO 6786:2001. The materials were placed in cylinders (measuring 15 mm in diameter and 5 mm in height) after mixing. Within 2 minutes after initiation of mixing, the materials with their cylinders were incubated at 37°C at a relative humidity of 95-100%. After 30 seconds to 1 minute, the special needle, measuring 2±0.1 mm, was placed on the surface of the material at a right angle to the surface using the Vicat penerometer (Schiller Park, Illinois, USA) at a crosshead speed of 1 mm/min and maintained in place for 5 seconds. The procedure was repeated every 30 seconds until the needle was unable to create a completely circular indentation on the material surface. The setting time was defined as the duration of time from the initiation of mixing until the needle was unable to create a completely circular indentation on the material surface.

-Working Time

This physical property was evaluated according to ISO 6786:2001. Appropriate amounts of the powder and liquid of each material were mixed for different durations (10 seconds and 10, 20, 30, 40 and 50 minutes); 0.5 mL of the mixture was placed at the center of a glass slab. Then another glass slab was placed on it with a weight of 100 g on it. This set was preserved in this state for 10 minutes. Then the diameter of the sample was measured. The working time was recorded when the diameter of the sample reached 10% of the original diameter.

-Compressive Strength

The compressive strengths were determined by using the ISO-9917 method. Each material was mixed and placed in a split stainless steel mold (measuring 4.0 mm inner in diameter and 6.0 mm in height). The cement was then compacted into each mold using a spatula and further compacted using a dental plugger to ensure a dense and uniform sample with minimal porosity. Once filled, the excess material was scraped off with the edge of a glass microscopic slide to leave a flat uniform surface. No later than 120 seconds after mixing, the complete assembly was transferred to a cabinet maintained at 37°C for 6 hours, after which they were removed from the molds and checked visually for any air voids or chipped edges.

Fifteen sound samples were prepared in each group and placed in closed containers that contained a gauze piece, impregnated with phosphate-buffered saline solution.

Compressive strength assessment was performed using a universal testing machine (Hounsfield Test Equipment, Model: H5K-S, Perrywood Business Park, Honey Corckland, Salfords, Redhill, Surrey, UK) 4 days after mixing. The device applied force at a crosshead speed of 1 mm/min in the direction parallel to the longitudinal axis of the molds until the materials were crushed. This force was recorded in Newton and converted to MPa by the following formula: CS=4p/μd2, where p is the maximum force applied in Newton’s and d is the mean diameter of the specimen in mm.

-Push-out Bond Strength

Thirty human maxillary central incisors were included in the present study. The inclusion criteria consisted of teeth with only one root canal, absence of previous root canal treatment and absence of any carious lesions. All the attached soft tissues were removed from the tooth surfaces with a periodontal curette (Hu-Friedy, Chicago, IL, USA) and the teeth were stored in 0.5% chloramine-T solution until the initiation of the study procedures. The tooth crowns were removed at cement-enamel junction (CEJ) level using a diamond disk (SP 1600 Microtome, Leica, Nu Block, Germany) and the working length (WL) was determined at 1 mm short of the apical foramen with a #15 K-file (Dentsply Maillefer, Ballaigues, Switzerland). The root canals were prepared using the crown-down technique with RaCe rotary system as follows: #40/0.10 and #35/0.08 for the coronal third, #30/0.06 for the middle third and #25/0.06 for preparation up to the WL. The irrigation protocol consisted of 2.5% NaOCl solution during instrumentation and a final flush with normal saline at the end of preparation procedures, followed by a 5-minute use of 17% ethylenediamine tetra acetic acid (EDTA) (Pulpdent Corporation, Watertown, MA, USA).

After preparation, the root canals were dried with paper points. Then 2 mm-thick root slices were prepared from the coronal third of the root canals, using a diamond saw (SP 1600 Microtome; Leica, Nu Block, Germany) and filled with MTA or MTA+TiO2.

Push-out test was carried out in a universal testing machine (Hounsfield Test Equipment, Model: H5K-S, Surrey, England). The force was applied in the apico-cervical direction of the samples at a crosshead speed of 1 mm/min. The maximum force (F) at bond failure was recorded in Newton. The push-out bond strength was calculated in MPa according to the following formula: F/2r×h (with r=3.14, r being the radius of the root canal and h being the thickness of the disk sample in mm).

-Statistical analysis

T-test was used to compare the means and standard deviations of the data on the physical properties of the experimental materials between the two groups in relation to the four properties evaluated. Statistical significance was set at *P*<0.05.

## Results

 Table 1 presents the means and standard deviations of the values in relation to the physical properties of the materials evaluated. In relation to all the 4 properties evaluated, t-test showed the significant effect of the material type (presence and absence of TiO2 nanoparticles) on the push-out bond strength, compressive strength, working time and setting time, with significantly higher values achieved in the group with TiO2 nanoparticles than the group without these particles (*P*=0.01 for the setting time and compressive strength, *P*=0.03 for the working time and *P*=0.001 for the bond strength).

**Table 1:**
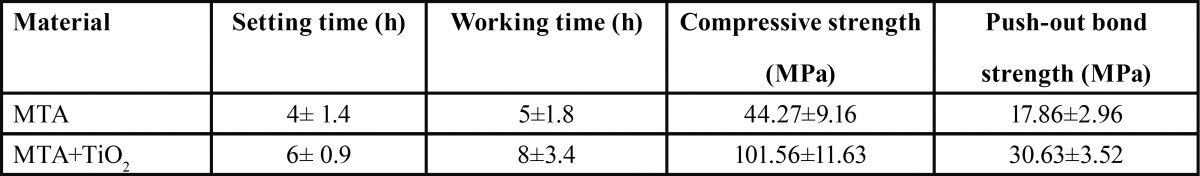
Mean± standard deviation of the evaluated properties of the tested materials.

## Discussion

The aim of the present study was to evaluate the effect of incorporating TiO2 nanoparticles into MTA on some of its physical properties (setting time, push-out bond strength, compressive strength and working time); the results showed significant increases in all the parameters mentioned above as a result of adding TiO2 nanoparticles to MTA.

MTA, was a biomaterial, is used in different endodontic procedures (perforation repair, root-end filling, pulpotomy and apexification) (3). On the other hand, attempts have always been made to improve and promote the physical properties of this material. In this context, a number of studies have evaluated the properties of MTA after incorporating different nanoparticles into its composition (2,29).

TiO2 nanoparticles, too, have recently attracted attention in the dental materials field. Incorporation of these nanoparticles to glass-ionomer and acrylic resin has resulted in a concentration-dependent improvement in the physical properties of these materials. In addition, mouthwashes containing these nanoparticles exhibit better antibacterial activity against *S. mutans* and *S. sanguis* (2). In a study, incorporation of these nanoparticles into Portland cement resulted in improvements in some of its physical properties (30). In the present study, similar to the study above, the wt% of titanium dioxide was 1% of the MTA powder.

Considering the clinical applications of MTA, the compressive strength of this material is very important in cases in which MTA is used for furcation repair, pulp capping and apexogenesis (13). In such cases, the material should resist against dislodgement due to the occlusal forces or placement of restorative materials over it. The comprehensive strength of hydraulic cements is considered an indication for its hydration and in fact it is an indirect reflection of the material’s setting reaction; this physical property is under the influence of the type of the MTA, the liquid it is mixed with, the condensation pressure and the technique used to mix the powder and the liquid (12,15).

In spite of the 4-hour setting time for MTA, it takes a few days for its compressive strength. to reach the maximum level, with primary compressive strength of 40 MPa after mixing rising up to 67 MPa within 21 days (15). Similar previous studies (12,15,16), a 4-day evaluation interval after mixing was selected in the present study to make it possible to compare the results.

Long setting time has always been considered as one of the disadvantages of MTA (3). On the other hand, after apical surgeries the retro-filling materials might be contaminated and washed away in contact with the influence of tissue fluids. Clinically, a setting time of 25-30 minutes is considered favorable (4). In fact, when a material sets fast there is a short time for its contamination in the oral cavity; on the other hand, the increase in initial strength, decreases the odds of its being washed away. As a result, the restorative material can safely be placed over it in the same session.

More specifically, any change in the setting process of bioactive materials, including the time and production of reaction products, which are mainly calcium and hydroxyl ions, might affect the production of hydroxyapatite layer and the bioactivity of these materials (13). The mixing technique, the amount of liquid used, the force used for packing and the environmental moisture affect the setting process (20). In the present study, all the variables were matched in all the samples except for the mixing technique. In this context, the powder-to-liquid ratio was 3:1 in all the samples; placement and packing of the materials were carried out by one operator and all the samples were stored under the same environmental conditions and moisture until used for the purpose of the study.

Considering the clinical applications of MTA, the bond strength of this material to dentin is an important factor in achieving a proper seal between the root canal and the external surface of the root (20). In other words, these materials should be able to resist dislodging forces, including functional forces or forces resulting from the placement of restorative materials, and retain their bond to dentin. Push-out bond strength test is a valuable technique for the evaluation of the bond between dental materials and dentin (20). After placement of MTA in the root canal, hydroxyapatite crystals nucleate and grow, filling the microscopic spaces between MTA and the dentinal wall. Initially this seal is mechanical. With time, the reaction between the hydroxyapatite layer and dentin leads to chemical bonding (25,26). Previous studies have shown that biomineralization of MTA significantly increases by exposure to phosphate buffered saline. Biomineralization takes place at MTA-dentin interface by formation of hydroxyapatite, and its deposition along the collagen fibers and the tag-like structures formed at the MTA-dentin interface (the interstitial layer) are responsible for the chemical bond (22). In the present study, resistance to dislodgment increased after incorporation of titanium dioxide nanoparticles.

## Conclusions

Under the limitations of the present study, incorporation of TiO2 into MTA had a positive effect on the push-out strength and compressive strength of MTA; however, it had a negative effect on the setting and working times. Therefore, further studies are recommended before its clinical application.
